# Supplementary material for: A new semi-automated, motility-based screening assay for discovery of compounds with activity against the juvenile stage of Fasciola hepatica
Source: PLoS Negl Trop Dis. 2026 Oct 26;20(10):e0014491. doi: 10.1371/journal.pntd.0014491 (PMC13722327; doi:10.1371/journal.pntd.0014491)
Supplement: Supplementary file 1 [file pntd.0014491.s002.pdf]

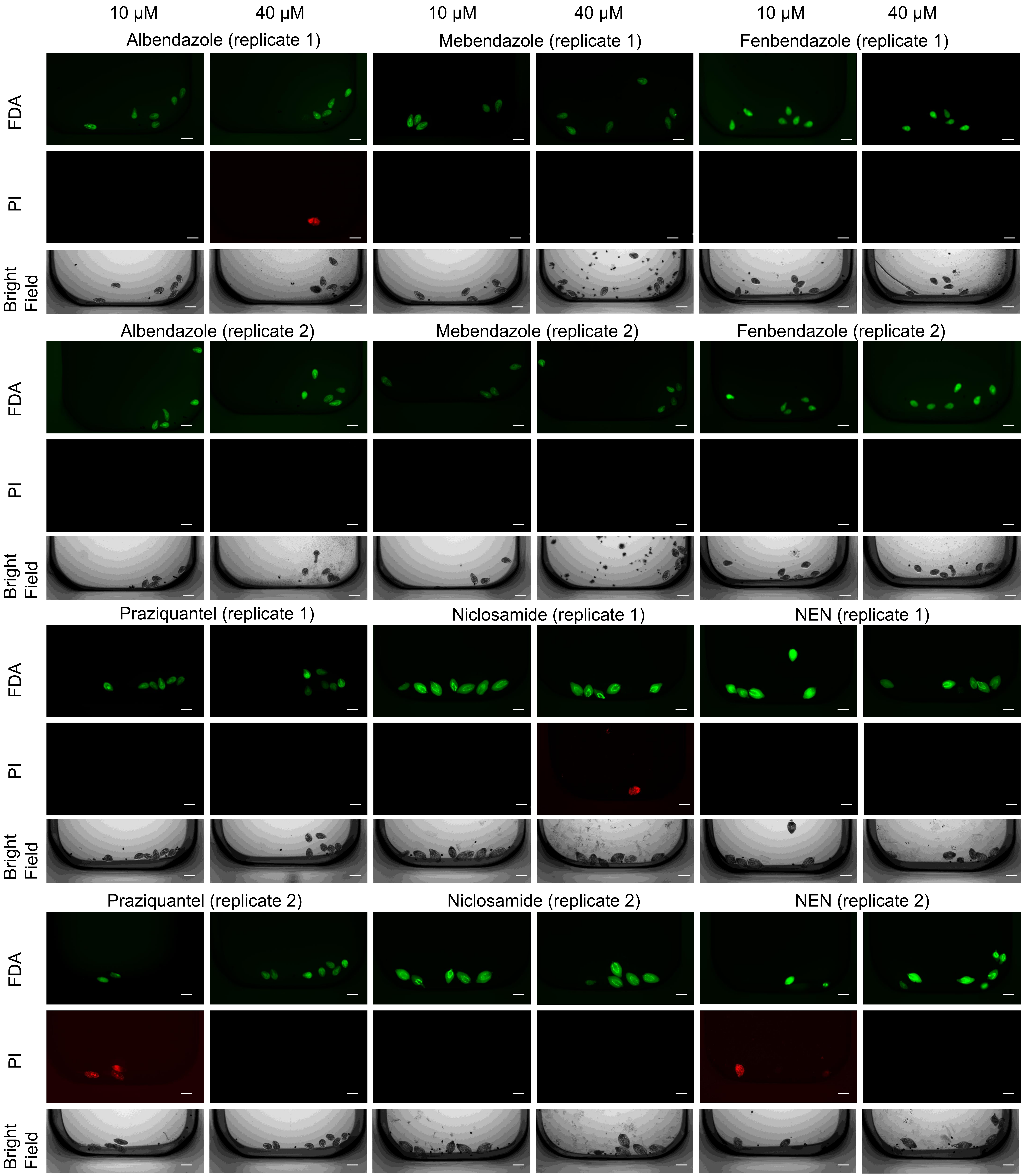

Figure S2: NEJ viability after drug exposure

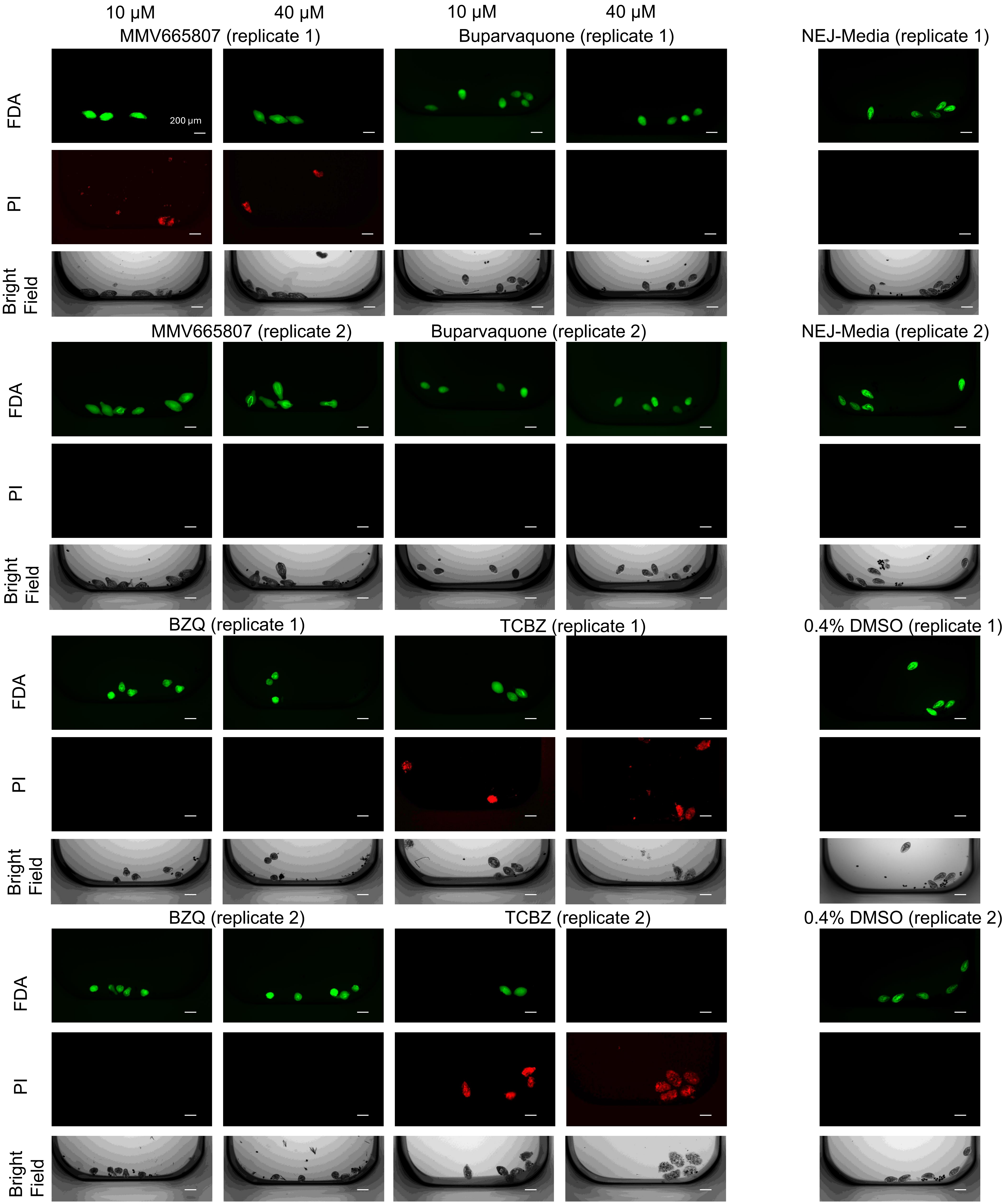

Figure S2: NEJ viability after drug exposure
